# An unusual case of bovine anthrax in the canton of Jura, Switzerland in 2017

**DOI:** 10.1186/s12917-019-1996-4

**Published:** 2019-07-29

**Authors:** Stefanie Gobeli Brawand, Sonja Kittl, Martina Dettwiler, Andreas Thomann, Simon Feyer, José Cachim, Grégoire Theubet, Nicole Liechti, Matthias Wittwer, Nadia Schürch, Simone Oberhänsli, Andreas Heinimann, Jörg Jores

**Affiliations:** 1 Institute of Veterinary Bacteriology, University of Bern, Bern, Switzerland; 2 Institute of Animal Pathology, University of Bern, Bern, Switzerland; 3 Animal Health, General Management of Agriculture, Viticulture and Veterinary Affairs (DGAV), Canton of Vaud, Switzerland; 4 Vétérinaires Mont-Terri Sàrl, Courgenay, Switzerland; 5 Spiez Laboratory, Federal Office for Civil Protection, Spiez, Switzerland; 6 Interfaculty Bioinformatics Unit, University of Bern, Bern, Switzerland; 7 Institute of Geography and Centre for Development and Environment, University of Bern, Bern, Switzerland

**Keywords:** Anthrax, Switzerland, Cave, Abortion, Bovine

## Abstract

**Background:**

Anthrax caused by *Bacillus anthracis* is a zoonotic disease mainly affecting herbivores. The last Swiss outbreak was over 20 years ago. We describe a recent anthrax outbreak involving two cows from the same herd. One cow was designated as a peracute clinical case with sudden death and typical lung lesions, while the other cow presented with protracted fever and abortion.

**Case presentation:**

On April 29th 2017, a 3.5-year-old Montbéliard dairy cow was found dead while out at pasture with haemorrhage from the nose. The veterinarian suspected pneumonia and performed a necropsy on site. Subsequently, a lung and liver sample were sent to the laboratory. Unexpectedly, *Bacillus anthracis* was isolated, a pathogen not found in Switzerland for decades. Several days later, a second cow from the same farm showed signs of abortion after protracted fever. Since these symptoms are not typical for anthrax, and the bacteria could not be demonstrated in blood samples from this animal, a necropsy was performed under appropriate biosafety measures. Subsequently, *Bacillus anthracis* could be isolated from the placenta and the sublumbal lymph nodes but not from the blood, liver, spleen and kidney. The outbreak strain (17OD930) was shown to belong to the lineage B.Br.CNEVA, the same as Swiss strains from previous outbreaks in the region. We speculate that the disease came from a temporarily opened cave system that is connected to an old carcass burial site and was flushed by heavy rainfall preceding the outbreak.

**Conclusion:**

Even in countries like Switzerland, where anthrax is very rare, new cases can occur after unusual weather conditions or ground disturbance. It is important for public officials to be aware of this risk to avoid possible spread.

## Background

Anthrax is caused by the Gram-positive bacterium *Bacillus anthracis.* It is primarily a disease of herbivores with high mortality [1]. *B. anthracis* forms spores outside the warm-blooded host after contact with oxygen and is therefore able to survive for decades in the environment. Bovines are usually infected through ingestion, or possibly inhalation, of spores when grazing on contaminated pastures [1]. After an incubation time of 1–14 days, normally, sudden death without any specific preceding symptoms is observed [1]. If the course of the disease is more protracted, as is more often the case in pigs or horses, fever and development of oedemas can be seen [1, 2]. Animals found dead on pasture, especially with haemorrhage from the mouth, nostrils or anus, should alert a veterinarian to the possibility of contracting anthrax [2].

Burial of the carcasses of diseased animals is contraindicated as it bears the risk of spore release, e.g. by physical soil disturbance at burial sites. In countries like Switzerland, where anthrax has not been observed in decades, new outbreaks can nevertheless occur if ancient burial sites are disturbed and thus spores released. Such outbreaks have also been described in Sweden [3].

**Fig. 1:**
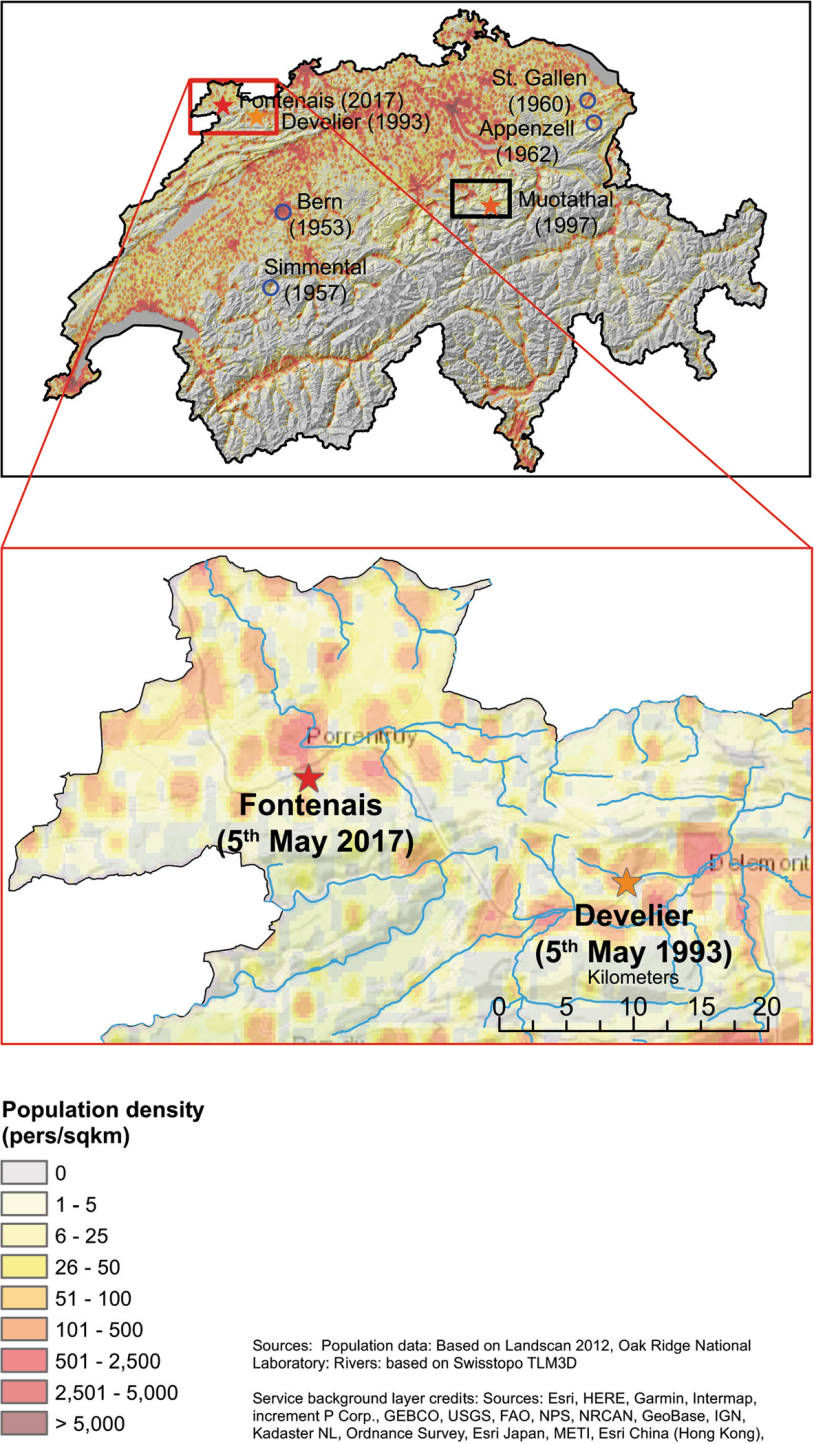
Population density in the region of the most recent bovine anthrax cases in the canton of Jura, Switzerland

**Fig. 2:**
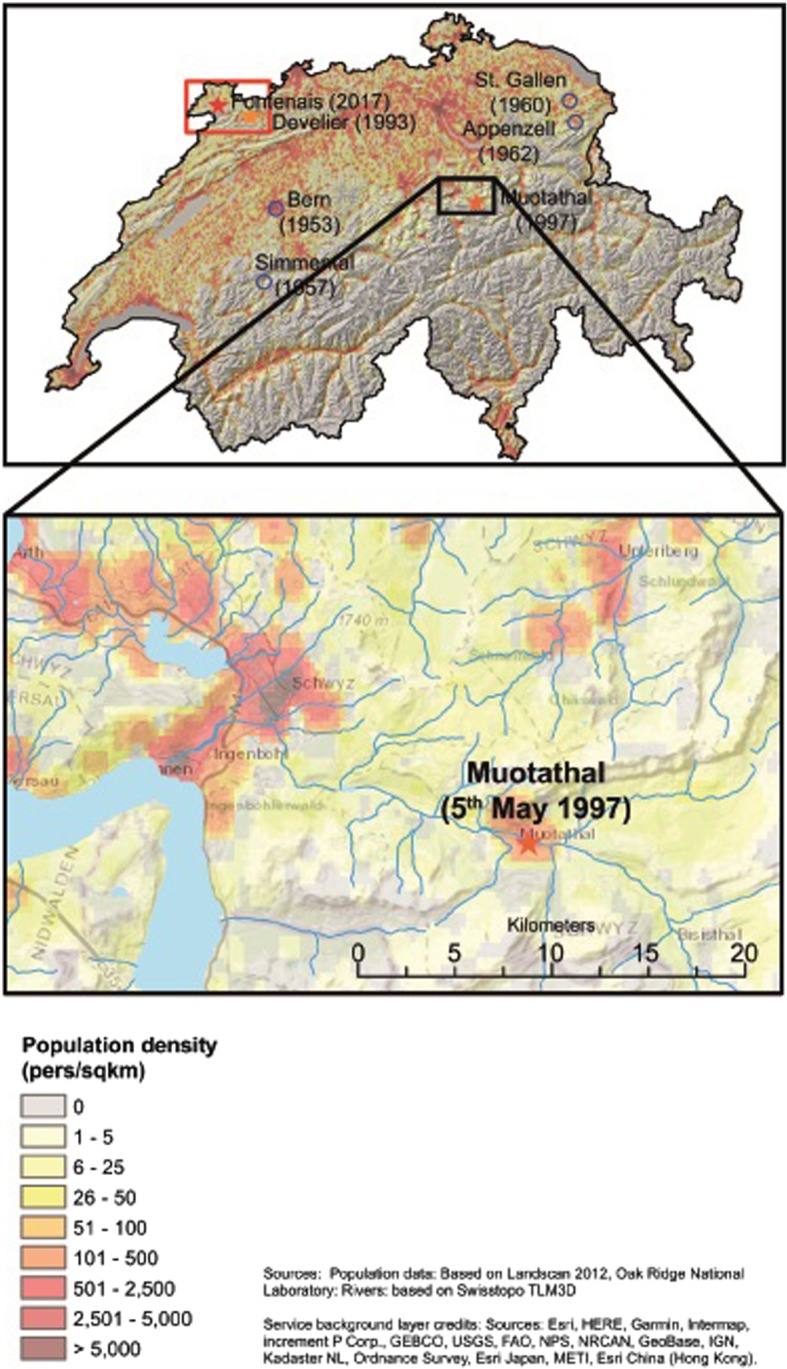
Population density in the region of the bovine anthrax cases in the Muotathal, canton of Schwyz, Switzerland

Furthermore, anthrax is a zoonosis transmitted mainly through direct or indirect contact with infected animals or by occupational exposure [1]. The last major anthrax outbreak in Switzerland occurred in 1985 in the canton of Grisons and caused the death of 11 cattle and 2 goats, but no human cases were reported from this outbreak [4]. However, there was a large outbreak in a wool factory in the North-eastern part of Switzerland lasting from 1978 to 1981, involving 25 workers who developed cutaneous or respiratory anthrax. The source of this outbreak was imported goat hair from Pakistan [5]. The last two cases of bovine anthrax in Switzerland date back to 1997 and 1993 and occurred in the municipality Muotathal (canton of Schwyz) and in the municipality Develier (canton of Jura), respectively (Figs. 1, 2).

We describe here an outbreak of bovine anthrax in Switzerland affecting two cows. The second case, where the animal showed protracted fever and signs of abortion, is an uncommon presentation of anthrax in cattle. Additionally, the suspected source of the outbreak – a temporary cave opening leading to a cave labyrinth connected to an old carcass dumping site – highlights the possibility of anthrax recurring in countries where it has not been observed for decades. As anthrax is a potentially fatal zoonosis, awareness among veterinarians and farmers is important, especially in regions where the disease is thought to be only of historical interest [6].

## Case presentation

On April 29th 2017, in the municipality of Fontenais in the canton of Jura (Fig. 1), in only 17 km air-line distance from the location of the 1993 outbreak, a 3.5-year-old Montbéliard dairy cow was found dead at pasture, showing dark haemorrhage from the nose. The veterinarian suspected pneumonia and dissected the animal on a trailer in a shed on the farm. Liver and lung were immediately sent to the Institute of Veterinary Bacteriology at the University of Bern, where *B. anthracis* was detected in lung tissue by microscopy and culture. Specifically, tissue material was Giemsa-stained for microscopic examination [7] and streaked onto trypticase soy agar plates containing 5% sheep blood (TSA-SB) (BD Becton Dickinson) following incubation at 37 °C for 18 h. Typical colonies were confirmed to be *B. anthracis* by a positive gamma phage lysis assay [8, 9] and by PCR, specific for the chromosomal marker *sap* and the plasmid markers *pag* and *cap* [10].

Consequently, the cantonal veterinary authority ordered a second-degree ban for this farm. Nine days later, nine bovines belonging to the same herd developed fever ranging from 39.5 to 40.2 °C. Venous blood samples from several affected animals were submitted for bacteriological analysis, where Giemsa-stain as well as a bacterial culture, performed as described above, were negative for *B. anthracis*. While eight animals recovered without any treatment, one animal, a pregnant 4.5-year-old cow, showed haemorrhagic vaginal discharge about 3 days after onset of fever. This cow was treated with a single dose of penicillin (Benzylpenicillinum procainum 20 mg/kg body weight intramuscularly; Procacillin®, MSD Animal Health GmbH, Luzern, Switzerland). The cantonal veterinary authorities asked for a necropsy of this animal under appropriate biosafety measures at the Institute of Animal Pathology (ITPA) of the University of Bern, as repeated blood samples taken before antibiotic treatment had tested negative, and clinical signs were unusual for an infection with *B. anthracis*. The diseased animal was transported to the Institute of Animal Pathology and euthanised 16 h after treatment with penicillin. Necropsy performed under special preventive measures [11] revealed macroscopically inconspicuous liver, spleen and kidneys, while the placenta showed haemorrhagic placentomes, and sublumbal lymph nodes were enlarged and necrotic. Giemsa-stained smears of the placenta and sublumbal lymph nodes revealed a massive yield of encapsulated rods, and culture followed by PCR confirmed the presence of *B. anthracis*. Resistance testing showed that the isolated strain was sensitive to penicillin.

In order to identify the potential source of infection, several people, including the farmer and his family, the cantonal veterinarian and a speleologist were interviewed on May 22, 2017. Based on the topographical, geographical and hydrological features, an outbreak hypothesis and a sampling plan were developed. The sampling scheme was based on the assumption that due to rainfall, anthrax spores were flushed out from a nearby cavity known as “trou des gez” and came up to the surface on the pasture where the outbreak occurred. A speleologist had dug up soil in this area while opening a small, historically known cave next to an old tree. According to his testimony, water spilt out and flowed over the grass area, whereupon he closed the cave again. This happened a few days prior to the onset of the outbreak. Soil, grass and water samples were taken from the farmland and in the cavity “trou des gez”, known to be a former burial site for many types of carcasses. In order to screen the farm infrastructure for contamination, swabs were taken from the trailer on which the first diseased cow had been necropsied and from the box in the barn where the second animal began to abort.

The sample analysis was performed at Spiez Laboratory using a modified protocol [12]. Briefly, 5 g of soil/grass was homogenised in PBS/Tween and mixed for several hours to dislodge spores. To settle solid particles, the samples were centrifuged at low speed (500 x *g*) for 30 s. To inactivate vegetative cells, the supernatant was heated at 80 °C for 30 min and subsequently centrifuged at 5,000 x *g* for 15 min to pellet spores and residual particles. To allow germination, the pellet was dissolved in 9 ml tryptic soy broth (TSB, BioMérieux) and incubated for 30 min at 37 °C. For PCR analysis, 1 mL was centrifuged at 5,000 x *g* for 15 min and the pellet was dissolved in AVL buffer (Qiagen). Prior to DNA extraction, the suspension was heated at 100 °C for 15 min followed by sterile filtration (0.45 μm, Millipore). Water samples were filtered through 0.45 μl filters and the filters, as well as the swabs, were eluted in PBS/Tween. Further steps were analogous to the soil samples. Real-time PCR analysis was performed as previously described. For PCR positive samples, serial dilutions of the TSB suspensions were streaked directly on cereus ID agar (Merck) for cultivation under BSL-3 (biosafety level 3) conditions. PCR analysis of the swab samples showed that *B. anthracis* spores were present on the trailer and in the shed where the first diseased cow was necropsied as well as in the box of the second affected cow. An isolate was retrieved from the trailer, whereas the soil and grass samples tested negative with the internal process controls confirming accurate sample preparation.

**Fig. 3:**
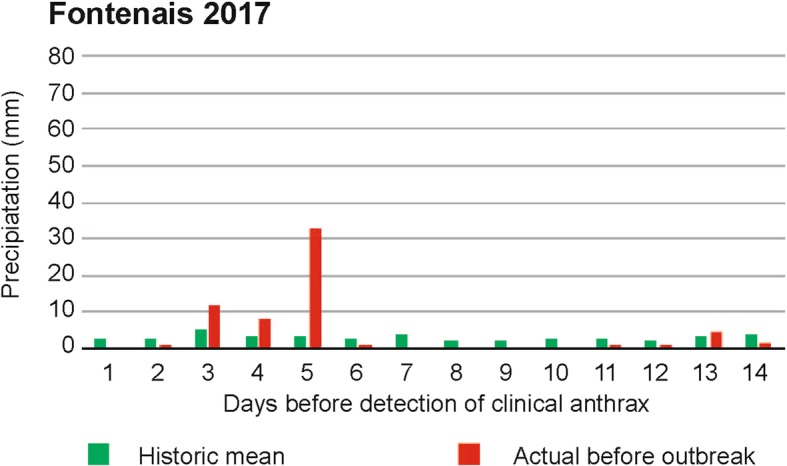
Daily rainfall amount preceding the most recent bovine anthrax case in Fontenais, Canton of Jura, Switzerland

**Fig. 4:**
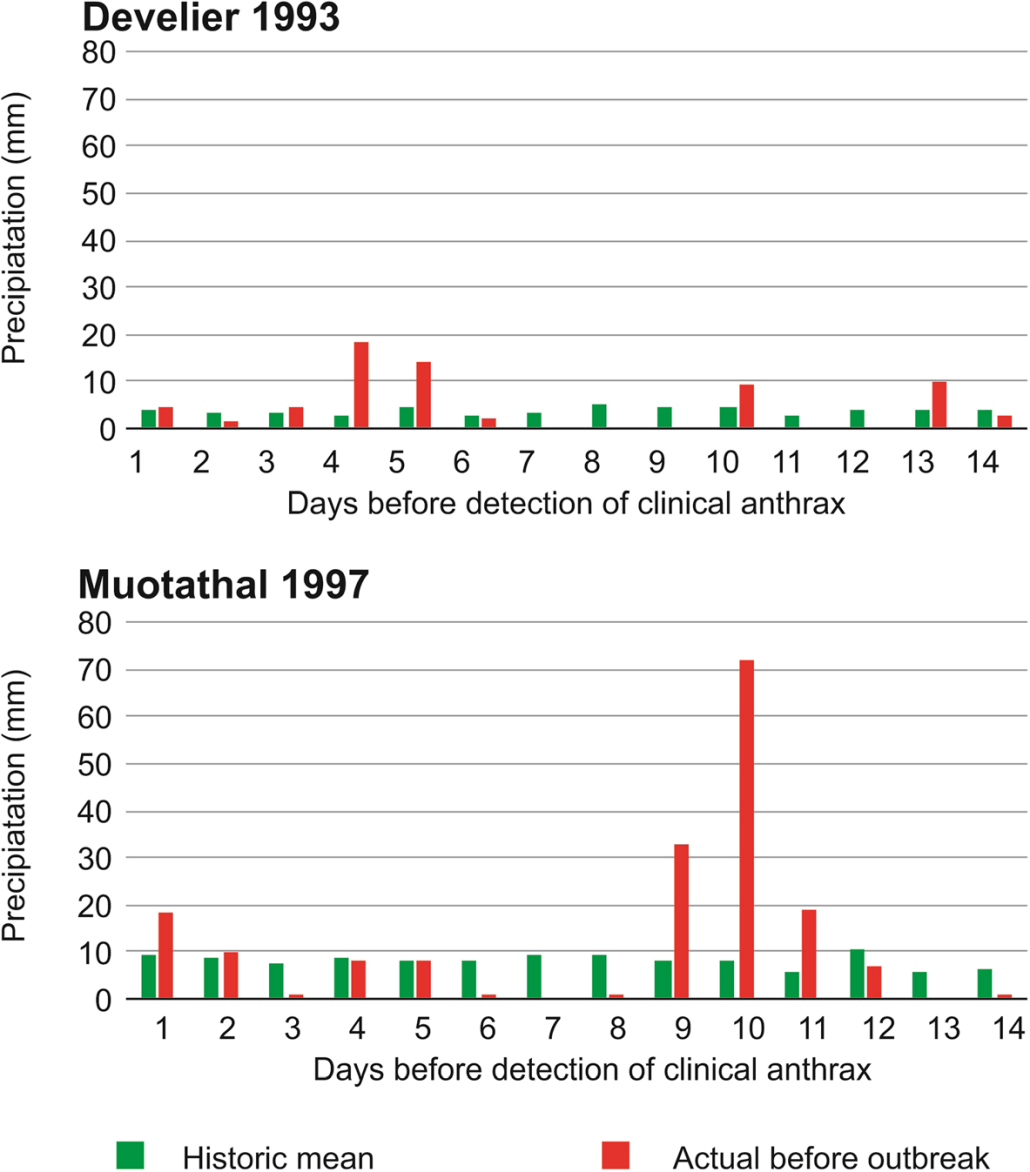
Daily rainfall amount preceding the bovine anthrax case in Muotathal, Canton of Schwyz, and Develier, canton of Jura, Switzerland

Daily rainfall patterns in the fourteen days preceding this case and the previous bovine anthrax cases in Muotathal and Develier were analysed and compared with the long-term daily mean from the years 1961 to 2016 using the RhiresD product [13]. The analysis revealed that in the 14-day-period before each case precipitation amounts were indeed higher than the long-term historic mean (+ 43% in Develier, + 75% in Muotathal and + 60% in Fontenais; Fig. 3, Fig. 4), substantiating the role of heavy rains ahead of soil disturbance.

To assess the potential risk to humans the population density around the outbreak area was determined based on Landscan 2012, Oak Ridge National Laboratory (population data) and on Swisstopo TLM3D (rivers). High population densities ranging between 500 and 2,500 persons living per km2 in a perimeter of 5 km around the 2017 outbreak location and the other two most recent Swiss bovine anthrax outbreak locations could be identified (Figs. 1, 2).

In order to sequence and subsequently compare the *B. anthracis* strain isolated in 2017 to other isolates from Switzerland and neighbouring countries, DNA was extracted following BSL-3 safety procedures. The strain was grown on TSA-SB at 37 °C for only 6 h to avoid production of spores. Extraction was performed using the QIAmp® DNA Blood Mini Kit (QIAGEN) using the manufacturer’s instructions for isolation of DNA for Gram-positive bacteria. The protocol was adapted as follows: bacteria were not pelleted, but a loop full was directly suspended in enzyme solution and was incubated at 56 °C overnight. The eluate was run through a 0.2 μm filter before transferring it to a BSL-2 lab, where the DNA was additionally concentrated by precipitation. Illumina paired-end sequencing (read length 250 bp) was performed by the Lausanne Genomic Technologies Facility (Lausanne, Switzerland) on a MiSeq platform. Approximately 13 Mio paired reads were obtained resulting in 600x coverage. The reads were quality controlled using fastqc [14] and then trimmed with trimmomatic 0.33 [15]. Reads were mapped to the Ames Ancestor (NC_007530.2) using the Burrows-Wheeler Aligner bwa 0.7.13 [16], followed by SNP (single nucleotide polymorphism) calling using samtools (1.3 and 0.1.19) [17]. For comparison, previously sequenced Swiss strains [18], as well as other published strains from all three clades [18], were also mapped to the same reference and SNPs called as described above. For the Swiss strains, reads were downloaded from the Sequence Read Archive while for the other strains, the assemblies were downloaded from GenBank and artificial reads created for mapping (for strain information and accession numbers see Table 1).

**Table 1:** Strains used for phylogenetic analyses sorted by clade [18]

Strain ID	Country of isolation	Year of isolation	Host species	GenBank Accession No	clade
9080G	Georgia	1998	soil	NZ_CM002398.1	A
A0193	USA	–	bovine	NZ_ABKF00000000.1	A
A0389	Indonesia	–	–	NZ_ABLB00000000.1	A
A0488	UK	1935	bovine	NZ_ABJC00000000.1	A
A1039	Bolivia	1999	bovine	NZ_LAKZ00000000.1	A
A1075	Chile	–	bovine	NZ_LBFE00000000.1	A
Ames Ancestor	USA	–	–	NC_007530.2	A
Australia94	Australia	1994	bovine	NZ_AAES00000000.1	A
Canada_bison	Canada	–	–	NZ_CP010322.1	A
JF3853	Switzerland	1952	bovine	ERR899845	A
PAK1	Pakistan	1978	ovine	NZ_CP009325.1	A
Turkey32	Turkey	1991	human	NZ_CP009315.1	A
Vollum	UK	1963	bovine	NZ_CP007666.1	A
17OD930	Switzerland	2017	bovine	SRP144421 (this study)	B
A0442	South Africa	–	–	NZ_ABKG00000000.1	B
A0465	France	1997	bovine	NZ_ABLH00000000.1	B
ANSES00–82	France	2000	bovine	NZ_JHDS00000000.2	B
BA1035	South Africa	–	human	NZ_CP009700.1	B
BF1	Germany	2009	bovine	AMDT00000000.1	B
CNEVA-9066	France	1992	bovine	NZ_AAEN00000000.1	B
HYU01	Korea	2009	soil	NZ_CP008846.1	B
JF3852	Switzerland	1953	bovine	ERR899844	B
JF3854	Switzerland	1957	bovine	ERR899846	B
JF3887	Switzerland	1960	bovine	ERR899847	B
JF3888	Switzerland	1962	bovine	ERR899848	B
K3	South Africa	–	human	NZ_CP009331.1	B
KrugerB	South Africa	–	–	NZ_AAEQ00000000.1	B
RA3	France	1998	bovine	NZ_CP009697.1	B
SVA11	Sweden	2011	bovine	NZ_CP006742.1	B
Zimbabwe89	Zimbabwe	–	–	NZ_JMPU00000000.1	B
2002013094	USA	1956	soil	NZ_CP009902.1	C

**Fig. 5:**
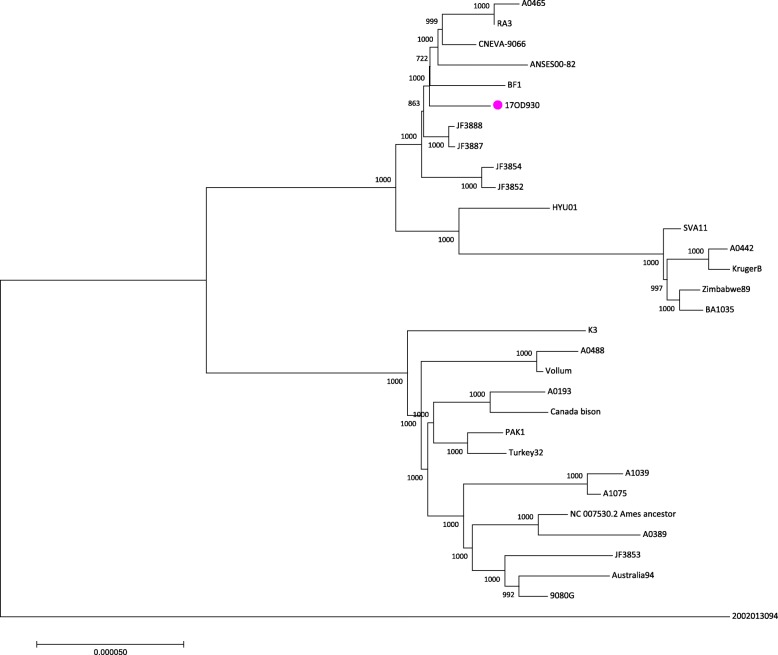
Whole genome SNP analysis comparing the 2017 outbreak strain to known strains of *B. anthracis*. The tree was constructed using PhyML with 1,000 replicates. The scale indicates the number of substitutions per site. The Clade C strain (2002013094) is used as outgroup

For the phylogenetic analyses, gap regions in the alignment were removed using MEGA7 [19] and the program modeltest-ng 0.1.3 (https://github.com/ddarriba/modeltest) was used to determine the best nucleotide substitution model. The program was run setting the topology parameter to maximum likelihood and otherwise using default parameters. The best model according to Bayesian information criteria was then chosen for phylogenetic tree construction with PhyML 3.3.20180214 [20, 21] and the tree was visualised in MEGA7. This whole genome SNP analysis revealed that *B. anthracis* strain 17OD930 clusters together with Swiss strains from prior outbreaks, as well as German and French strains (all of the B.Br.CNEVA lineage [18]). However, 17OD930 is not specifically closely related to any of the Swiss strains as compared to other strains in the same cluster. This indicates that it is not directly linked with earlier outbreaks strains that have been sequence characterised here (Fig. 5).

Additionally, de novo assembly was performed using SPAdes 3.10.1 [22]. Fourteen contigs were obtained for the chromosome and their locations determined by mapping to the Ames Ancestor (NC_007530.2). Gaps were bridged by read-mapping to the Ames ancestor and the RA3 strain (NZ_CP009697.1). Ambiguities were resolved by Sanger sequencing. The closed genome sequence was then submitted to GenBank (GenBank accession No: CP029323).

The direct comparison of both isolates from this recent outbreak (retrieved from the trailer where the first cow was necropsied, and the isolate from the second cow) shows as expected identical sequencing data which confirms that both cows were infected by the same genotype, i.e. strain.

While the above-mentioned analyses were conducted, the cantonal authorities decreed measures to contain the outbreak. As stated before, the cantonal veterinary office immediately ordered a so-called second-degree ban upon being informed of the positive anthrax result. The ban included restrictions on the movement of animals and persons living on the farm. Temperature measurements of all animals were conducted twice daily. Two neighbouring sheep farms were instructed to observe their animals for clinical signs. The cantonal office of public health together with the cantonal veterinary office surveyed the health of the involved humans. Nasal swabs analysed by PCR were negative. The federal office of public health, as well as the federal food safety and veterinary office, were also informed and the latter announced the case to the OIE. The public was informed by means of a press release. Epidemiological investigations to discern the source of infection were conducted as described, and the small recently human-made cave opening extending to an old tree on the pasture was firmly closed again. Decontamination of the barn, its annexes, the quarantine box and the milking room, as well as the shed and trailer, was conducted by the cantonal veterinary service in collaboration with the cantonal fire brigade using 10% formalin solution followed by 1% peracetic acid and hydrogen peroxide for final disinfection. The manure heap was treated by self-heating up to 70 °C for four days.

## Discussion and conclusions

Anthrax in cattle manifests as a severe disease with a fulminant course ending in sudden death in most cases. A protracted course followed by abortion as described here is unusual in this species [2]. The disease process fits the unusual findings of the necropsy, which revealed a macroscopically and bacteriologically inconspicuous spleen. Usually, this organ shows a dramatic macroscopic change in cases of infection with *B. anthracis* [2]. This case confirms, that anthrax spores are still present in Switzerland and infections may take unexpected clinical courses. In this unusual anthrax outbreak the responsible veterinarian did not anticipate an infection with *B. anthracis* and therefore opened the carcass which poses a risk of contracting anthrax. The veterinarian was consequently treated with antibiotics and did not develop disease. The very low prevalence of anthrax in Switzerland contributes to a limited awareness of the disease among practising veterinarians. It is very important to include anthrax infection as a differential diagnosis when the sudden death of cattle at pasture occurs and to treat carcasses of such animals accordingly to minimise the risk of infection and spread of the pathogen. Furthermore, this outbreak clearly showed that different animals from the same herd infected with anthrax can present very different clinical outcomes including unusual placentitis. In addition, population density around the sites of the last bovine anthrax cases was shown to be high, underlining the possible risk these bovine anthrax cases pose to the human population. Informing the public properly and swiftly helps to prevent human infections in the area of the outbreak. Medical doctors should also keep anthrax on their list of differential diagnosis in regions that experienced anthrax outbreaks even a long time ago.

Although *B. anthracis* is usually susceptible to penicillin [1], *B. anthracis* could still be detected in the organs of the diseased animal. The isolate tested sensitive for penicillin and no antibiotic resistance genes or point mutations were detected that could substantiate the isolation of live bacteria after antibiotic treatment. A possible explanation may be that the interval of 16 h between treatment and euthanasia was too short for the antibiotic to eliminate the bacteria or that the drug did not pass the barrier between bloodstream and placenta because the process of abortion was already too advanced.

Although numerous environmental samples were taken, the source of the infection could not be determined. Nevertheless, it is plausible that the water pouring from the recent cave opening next to an old tree contaminated a limited area of the pasture where the cows gathered for milking at the farm. Grazing and animal movement through this highly frequented area of the pasture may have dispersed and diminished the spore concentration. The rather atypical manifestation of the disease in the lung is in accordance with a superficial deposition of the spores on the vegetation by water flow and subsequent inhalation during grazing.

Data from rainfall analysis could not be evaluated statistically, however, there were some rainy days prior to the outbreak, which may have led to the flush out of anthrax spores from the described cave labyrinth in the karst landscape. Unfortunately, no isolates of past anthrax outbreaks in the Canton of Jura were available for genetic comparison. The strains from other former Swiss outbreaks did not show any closer relation to our strain than isolates from surrounding countries and have therefore no correlation with the described outbreak.

## Data Availability

The genome sequence data generated during the current study are available in the GenBank repository under Accession No. CP029323.1 (genome) and SRP144421 (reads). BioProject PRJNA454788.

## Abbreviations

BSL-3: Biosafety level 3
SNP: Single nucleotide polymorphism
TSA-SB: Trypticase soy agar plates containing 5% sheep blood
TSB: Tryptic soy broth
